# Hormone-induced sperm-release in the critically endangered Booroolong frog (*Litoria booroolongensis*): effects of gonadotropin-releasing hormone and human chorionic gonadotropin

**DOI:** 10.1093/conphys/coy080

**Published:** 2019-02-05

**Authors:** Aimee J Silla, Michael S McFadden, Phillip G Byrne

**Affiliations:** 1 School of Earth, Atmospheric and Life Sciences, University of Wollongong, Wollongong, NSW, Australia; 2 Herpetofauna Department, Taronga Conservation Society Australia, Mosman, NSW, Australia

**Keywords:** Amphibian, captive breeding, conservation, gamete release, reproduction, reproductive technologies, spermiation

## Abstract

Research into the development of reproductive technologies for amphibians has increased in recent years due to the rapid decline of amphibian species globally. Reproductive technologies have great potential to overcome captive breeding failure and improve the propagation and genetic management of threatened species. However, the incorporation of these technologies into conservation breeding programs has been protracted, primarily as a result of trial-and-error approaches to the refinement of hormone therapies. The present study investigated the effects of: (1) GnRH-a dose (0, 0.5, 1, 2, 4, 8 or 16 μg g^−1^), and (2) hCG dose (0, 2.5, 5, 10, 20 or 40 IU g^−1^), on the sperm-release response of the critically endangered Booroolong frog. Administration of GnRH-a at a dose of 0.5 μg g^−1^ resulted in the greatest number of sperm released (mean total sperm = 3.5 ×10^6^, *n* = 11). Overall, hCG was more effective at eliciting spermiation in Booroolong frogs, with peak sperm release (mean total sperm = 25.1 ×10^6^, *n* = 10) occurring in response to a dose of 40 IU g^−1^. Sperm output in response to 40 IU g^−1^ hCG was greatest between 1 and 6 h and steadily declined between 8 and 24 h post-hormone administration. Percent sperm motility peaked between 4 and 10 h (58.1–62.7%), and sperm velocity between 4 and 12 h (24.3–27.2 μm s^−1^). Booroolong frogs join a small, but growing number of amphibian species that exhibit improved spermiation in response to hCG. Further research is required to identify optimal hormone-induction protocols for threatened amphibians and expedite the incorporation of reproductive technologies into CBPs.

## Teaser

Reproductive technologies have great potential to overcome captive breeding failure and improve the propagation and genetic management of threatened species. Here we report on the development of hormone therapies to induce sperm-release in the critically endangered Booroolong frog, *Litoria booroolongensis*.

## Introduction

Ongoing species decline and extinction continues to threaten vertebrate biodiversity across all taxonomic groups and geographic locations globally (Ceballos *et al.*, 2015). Among vertebrates, amphibians have experienced an exceptionally rapid loss of biodiversity, with at least 2100 species at imminent risk of extinction, representing 31.8% of all described species (IUCN, 2017). In response to this extinction crisis, conservation breeding programs (CBPs) have been established for a number of threatened amphibian species with some evidence of successful reintroduction and species recovery (Harding *et al.*, 2016). Despite such celebrated triumphs, disparity remains between the scale of the global amphibian crisis and the response from the conservation community, with current estimates indicating that a mere 2.9% (213 species) of all amphibian species have been the subject of CBPs (Harding *et al.*, 2016). One of the major challenges hindering the success of CBPs, and limiting the establishment of new programs, is captive breeding failure. Numerous reports document an inability to reliably and predictably initiate amphibian reproductive behaviour, achieve high rates of fertilisation, or generate viable offspring in a captive environment (Kouba *et al.*, 2009). Reproductive failure threatens the genetic viability and adaptive capacity of captive colonies and in many cases has limited the generation of large-numbers of individuals for reintroduction (Silla and Byrne, 2019). Reproductive technologies have the potential to contribute to threatened species recovery by allowing the manipulation of the neuroendocrine system to control reproductive events (Silla and Byrne, 2019; Vu and Trudeau, 2016). When used in concert with traditional captive breeding methods, reproductive technologies may improve the efficiency and sustainability of CBPs and enhance program success (Silla and Byrne, 2019).

At present, two hormones dominate the literature as those most frequently employed to stimulate gamete release and spawning in amphibians; purified human chorionic gonadotropin (hCG) and synthetic gonadotropin-releasing hormone (GnRH-a, also known as luteinizing hormone-releasing hormone, LHRH-a). Exogenous hCG mimics the structure and bioactivity of natural luteinizing hormone (LH) molecules, simulating the LH surge required to stimulate gamete maturation and release by binding to LH-receptors on the gonads (hypophyseal approach; Silla and Byrne, 2019; Vu and Trudeau, 2016). In contrast, GnRH-a acts at a higher level of the hypothalamic-pituitary-gonadal (HPG) axis, triggering the synthesis and release of natural LH and Follicle Stimulating Hormone (FSH) from the anterior pituitary, which then promotes gonadal activity (hypothalamic approach; Silla and Byrne, 2019; Vu and Trudeau, 2016). The administration of GnRH-a is generally regarded as more effective at eliciting a consistent and predictable gamete-release response across a diversity of species (Kouba *et al.*, 2009; Silla and Byrne, 2019). However, a growing number of amphibians are reported to respond more favourably to hCG, including several species from the Bufonid, Limnodynastid and Pelodryadid families (Kouba *et al.*, 2012; Clulow *et al.*, 2018; Silla and Roberts, 2012). The inconsistent response to hCG administration is thought to be driven by interspecific differences in LH-receptor affinities (Clulow *et al.*, 2014), which may reflect the divergent evolution of affinities of LH-receptors among amphibian families (Silla and Roberts, 2012). At present, insufficient data exists directly comparing the gamete-release response of amphibians from different lineages to the administration of GnRH-a and hCG. Consequently, our capacity to predict the response of novel species is poor, and hormonal induction protocols need to be developed on a species-specific basis.

Central to the development of hormonal induction protocols for novel species is the establishment of dose-response curves for each hormone type (GnRH-a and hCG), and verification of optimal gamete-collection times post-administration (PA). If suboptimal or supraoptimal hormone doses are administered, gamete release will not occur or gametes will be released, but in lower quantities and with reduced viability and fertilisation capacity (Silla, 2010; Silla *et al.*, 2018). Optimal doses required to elicit gamete release have been shown to vary enormously among amphibian species. In particular, effective hormone doses used to induce sperm release have been reported to vary from 0.3 to 5 μg g^−1^ body weight GnRH-a and 13 to 40 IU g^−1^ body weight hCG (hormones administered via injection; Silla and Byrne, 2019). In addition to species-specific variation in optimal doses, amphibian species vary considerably in the speed and length of time in which they respond to hormone stimulation (Silla and Byrne, 2019). For example, male *Crinia georgiana* respond to the administration of GnRH-a within minutes (peak sperm release = 7 h PA; (Silla and Roberts, 2012)), while male *Pseudophryne corroboree* do not respond for several hours (peak sperm release = 36 h PA (Byrne and Silla, 2010)).

To date, the development of hormonal induction protocols for novel species has mostly involved a ‘trial-and-error’ approach, which can take years to refine (Kouba *et al.*, 2009; Silla and Byrne, 2019). This approach is hindering the incorporation of reproductive technologies into endangered species CBPs, where there is a race against time to implement successful conservation actions (Silla and Byrne, 2019). The present study aimed to empirically test protocols to hormonally induce spermiation in the critically endangered Booroolong frog, *Litoria booroolongensis*. Specific objectives were to investigate: (1) the effect of GnRH-a dose (0, 0.5, 1, 2, 4, 8 or 16 μg g^−1^ GnRH-a), and (2) the effect of hCG dose (0, 2.5, 5, 10, 20 or 40 IU g^−1^ hCG), on the number of spermiating males and the total number of sperm released. Additionally, the optimal hormone type and dose identified was used to investigate the effect of sampling time on the number of sperm released, and the effects of both sampling time and sample dilution on sperm quality (motility and velocity).

## Material and methods

### Ethics statement

Procedures outlined herein were conducted following evaluation and approval by the University of Wollongong’s Animal Ethics Committee (protocol numbers AE11/23 and AE12/17).

### Study species

**Figure 1:**
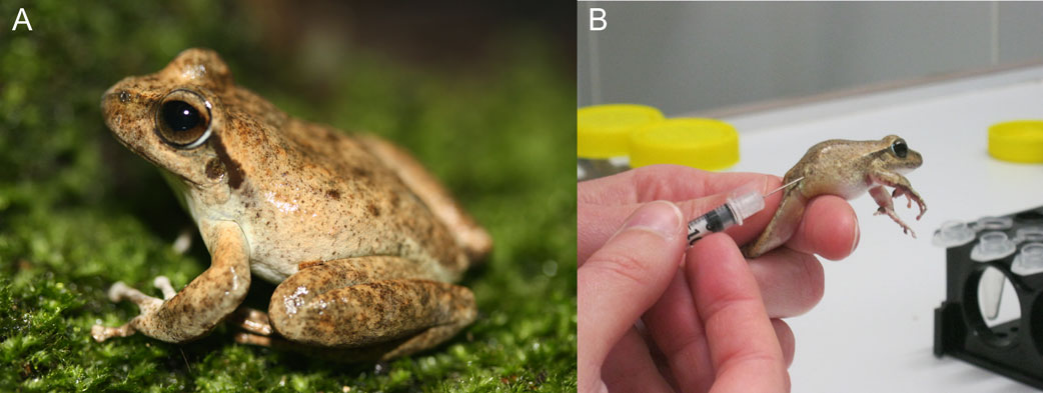
(**A**) Adult male Booroolong frog, *Litoria booroolongensis*, (**B**) subcutaneous injection of reproductive hormones.

The Booroolong frog, *L. booroolongensis* is a medium sized (36–54 mm snout vent length) riverine species with mottled brown, olive or grey dorsal markings (Fig. 1a; Tyler and Knight, 2011). *L. booroolongensis* is endemic to the Great Dividing Range of south-eastern Australia at an altitudinal range of 200−1300 m above sea level (Hunter and Smith, 2013). This species is associated with permanent streams characterised by riparian rocky habitat and an abundance of submerged smooth rock crevices (Hunter and Smith, 2013). *L. booroolongensis* breeds during austral spring to early summer, from October to early January (Hunter, 2007). During the breeding season, males advertise from exposed rock platforms and egg deposition occurs within shallow isolated rock pools or along slow-flowing sections of stream (Anstis, 2013). Females deposit a large clutch of 688–1784 eggs (average = 1296) adhered to aquatic rock crevices (Anstis, 2013). *L. booroolongensis* is currently listed as endangered by state and federal legislation in Australia and critically endangered by the International Union for Conservation of Nature (Hunter and Smith, 2013).

### Animals

Booroolong frogs were reared until sexual maturity at Taronga Zoo (Sydney, NSW, Australia). Frogs were first generation (F1) captive-bred individuals from field-caught parents (collected from the Abercrombie and Retreat River regions, 34° 7′S, 149° 38′E). Males were transported to the Ecological Research Centre at the University of Wollongong (Wollongong, NSW, Australia), where they were maintained according to husbandry methods outlined previously (Silla *et al.*, 2015). Prior to the initiation of experiments, frogs were determined to be in breeding condition by the darkening of their nuptial pads and initiation of calling behaviour. Frogs (*n* = 135) were 1–2 years of age during the study period and ranged in mass from 3.20 to 7.52 g (mean ± SEM = 4.73 ± 0.092 g).

### Experiment 1: the effect of GnRH-a dose

To determine the effect of GnRH-a dose on spermiation, 75 male frogs were allocated to one of seven experimental treatments; 0, 0.5, 1, 2, 4, 8 or 16 μg g^−1^ body weight GnRH-a (Leuprorelin acetate; Lucrin®) (*n* = 8−12 per treatment). A urine sample was collected from each male prior to the administration of hormones and in all cases the sample was aspermic. Hormones were diluted in 100 μl of Simplified Amphibian Ringer (SAR; 113 mM NaCl, 2 mM KCl, 1.35 mM CaCl2, 1.2 mM NaHCO3). Frogs received a single hormone dose, corresponding to their experimental treatment, injected subcutaneously using ultra-fine 31-gauge needles (Fig. 1b). Immediately prior to hormone injection, frogs were weighed to the nearest 0.01 g and the dose administered was adjusted according to an individual’s body mass.

Post-hormone injection, frogs were placed in individual vials containing three pieces of sponge (40 × 40 × 3 mm) wetted with 5 ml of RO water. Hydrating individuals according to this procedure was essential in order to permit spermic urine collection at each of the sampling times (1, 2, 4, 6 and 8 h post-hormone injection). Spermic urine was collected, and sample volume measured, according to protocols described previously (Silla, 2010, 2011). At each collection period, sperm concentration was determined using an Improved Neubauer Haemocytometer (Bright Line, Optik Labor, Germany). Spermic urine samples were homogenised and a 15-μl aliquot was pipetted into the haemocytometer chamber. The number of sperm present in five quadrats was recorded (repeated twice per sample and averaged) and used to calculate total sperm concentration per microlitre. The total number of sperm released was then calculated by multiplying sperm concentration (sperm μl^−1^) by spermic urine volume (μl). The total number of sperm released per individual was the sum of the number of sperm released at each sampling period (1, 2, 4, 6 and 8 h). Experiment 1 was conducted from 19 September to 6 October 2011, during the species’ natural breeding season.

### Experiment 2: the effect of hCG dose

To determine the effect of hCG dose on spermiation, 50 male frogs were allocated to one of six experimental treatments; 0, 2.5, 5, 10, 20 or 40 IU g^−1^ body weight hCG (Chorulon®) (*n* = 8–10 per treatment). A urine sample was collected from each male prior to the administration of hormones and in all cases the sample was aspermic. Hormones were diluted in 100 μl of SAR and injected subcutaneously using ultra-fine 31-gauge needles (Fig. 1b). Immediately prior to hormone injection, frogs were weighed to the nearest 0.01 g and the dose administered was adjusted according to an individual’s body mass. Spermic urine was collected at 1, 2, 4, 6 and 8 h post-hormone injection and the total number of sperm released per. individual was determined according to the procedures outlined in ‘experiment 1’ above. Experiment 2 was conducted from 30 October to 26 November 2012, during the species’ natural breeding season.

### Experiment 3: the effects of sampling time and sample dilution

To determine the effect of sampling time on the number of sperm released, and the effects of sampling time and sample dilution on the number of sperm released, sperm motility and sperm velocity, 10 male frogs were injected with 40 IU g^−1^ body weight hCG (Chorulon®). Post-hormone injection, frogs were placed in individual vials containing three pieces of sponge (40 × 40 × 3 mm) wetted with 5 ml of RO water every 8 h. Hydrating individuals according to this procedure was essential in order to permit spermic urine collection at each of the sampling times (0, 1, 2, 4, 6, 8, 10, 12, 14, 16, 18, 20, 22 and 24 h post-hormone injection). Spermic urine was collected at each sampling time according to protocols described previously (Silla, 2010, 2011). The total number of sperm released was determined for each individual at each sampling time. Additionally, percent sperm motility and sperm velocity (VCL: curvilinear velocity) was quantified at each sampling time using a computer-assisted sperm analysis system (CASA: CEROS version 12; Hamilton Thorne, Beverley, MA). Spermic urine samples were homogenised and two 10-μl aliquots were removed, the first aliquot was assessed undiluted, while the second aliquot was diluted in 10 μl of 1:16 SAR prior to assessment. Sperm motility (%) and sperm velocity (μm s^−1^) were recorded following a settlement period according to the procedures of Silla *et al.* (2015) and Keogh *et al.* (2017b), whereby the suspension was pipetted into a haemocytometer chamber (exact depth 0.1 mm) and placed on the microscope stage for 2 min to allow fluid to settle prior to analysis. Four replicate recordings were taken and averaged. Sperm performance parameters were measured in a constant temperature room set to 22°C. The CASA system used for the assessment of sperm motility and velocity was fixed according to settings described previously (Keogh *et al.*, 2017a; Silla *et al.*, 2017). Experiment 3 was conducted from 6 December to 10 December 2012, during the species’ natural breeding season.

### Statistical analyses

The number of males spermiating was compared between GnRH-a dose treatments and hCG dose treatments using two-tailed Fisher’s exact tests. To assess the effect of GnRH-a dose and hCG dose on the total number of sperm released, two separate non-parametric Kruskal–Wallis tests were conducted. Within each model, the response variable was total sperm released and treatment was a fixed factor. In order to control for body size effects, analyses were based on the residuals from linear regressions of total sperm number against male body mass. Post hoc treatment comparisons were made using Wilcoxon matched-pair tests.

To assess the effect of sampling time on the number of sperm released, a linear mixed effects (LME) model fitted with restricted maximum likelihood (REML) was performed, where sampling time was a fixed categorical effect, male ID was a random effect, and the response variable was total sperm released. To asses the effects of sampling time and sample dilution on sperm motility (%) and sperm velocity (VCL: μm s^−1^), two separate LME models fitted with REML were performed. Within each model, sampling time and dilution treatment (undiluted or diluted) were fixed categorical effects, male ID was a random effect and the response variable was either percent motility or sperm velocity. Prior to analysis, all percentage motility data were arc sine transformed using the transformation sin^−1^(√x). Body mass was not included in any of the LME models presented as body mass did not have a significant effect on any of the response variables (total sperm, sperm motility or sperm velocity) when included in the model as a random effect. All statistical analyses were performed using JMP Pro 11.0.0 software package (SAS Institute Inc. North Carolina, USE). For all analyses, statistical significance was accepted at *P* < 0.05.

## Results

### Experiment 1: the effect of GnRH-a dose

**Figure 2:**
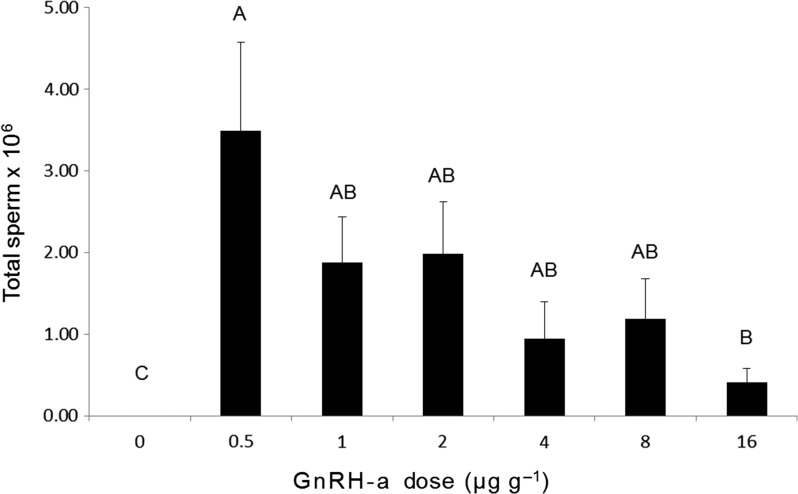
Effect of GnRH-a dose on the total number sperm released. Data are shown as the mean ± SEM (*n* = 8–12 per treatment). Letters displayed are the result of Wilcoxin post hoc tests. Treatments that share a letter are not significantly different (*P* > 0.05).

The number of frogs spermiating in response to the administration of 0 μg g^−1^ GnRH-a (0 %) was significantly lower than all other dose treatments (Fisher’s Exact tests, *P* < 0.05), which did not differ significantly from one another (0.5, 1, 2, 4, 8 or 16 μg g^−1^ GnRH-a; 63–100%; Fisher’s Exact tests, *P* > 0.05). Overall, the total number of sperm released differed significantly among treatment groups (Kruskal–Wallis test, *χ*^2^ = 13.708, *P* = 0.0331), with mean total sperm released significantly higher (Wilcoxin, *P* < 0.05) in the 0.5 μg g^−1^ GnRH-a dose treatment compared with the 0 and 16 μg g^−1^ GnRH-a dose treatments (Fig. 2).

### Experiment 2: the effect of hCG dose

**Figure 3:**
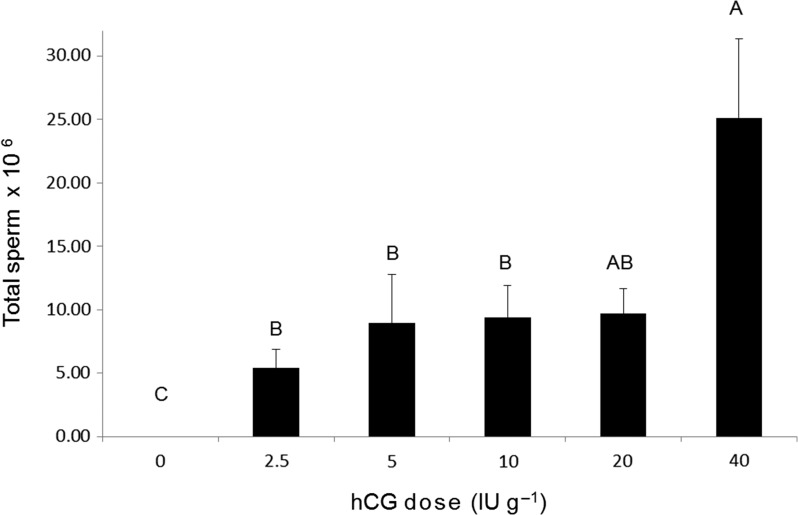
Effect of hCG dose on the total number of sperm released. Data are shown as the mean ± SEM (*n* = 8–10 per treatment). Letters displayed are the result of Wilcoxin post hoc tests. Treatments that share a letter are not significantly different (*P* > 0.05).

The number of frogs spermiating in response to 0 IU g^−1^ hCG (0%) was significantly lower than the number of frogs spermiating in response to all other treatments (2.5, 5, 10, 20 or 40 IU g^−1^ hCG; 100 %; Fisher’s Exact tests, *P* < 0.05). Overall, the mean total number of sperm released differed significantly among treatment groups (Kruskal–Wallis test, *χ*^2^ = 19.454, *P* = 0.0016), with mean total sperm released significantly higher (Wilcoxin, *P* < 0.05) in the 40 IU g^−1^ hCG dose treatment compared with the 0, 2.5, 5 and 10 IU g^−1^ hCG dose treatments (Fig. 3).

### Experiment 3: the effects of sampling time and sample dilution

**Figure 4:**
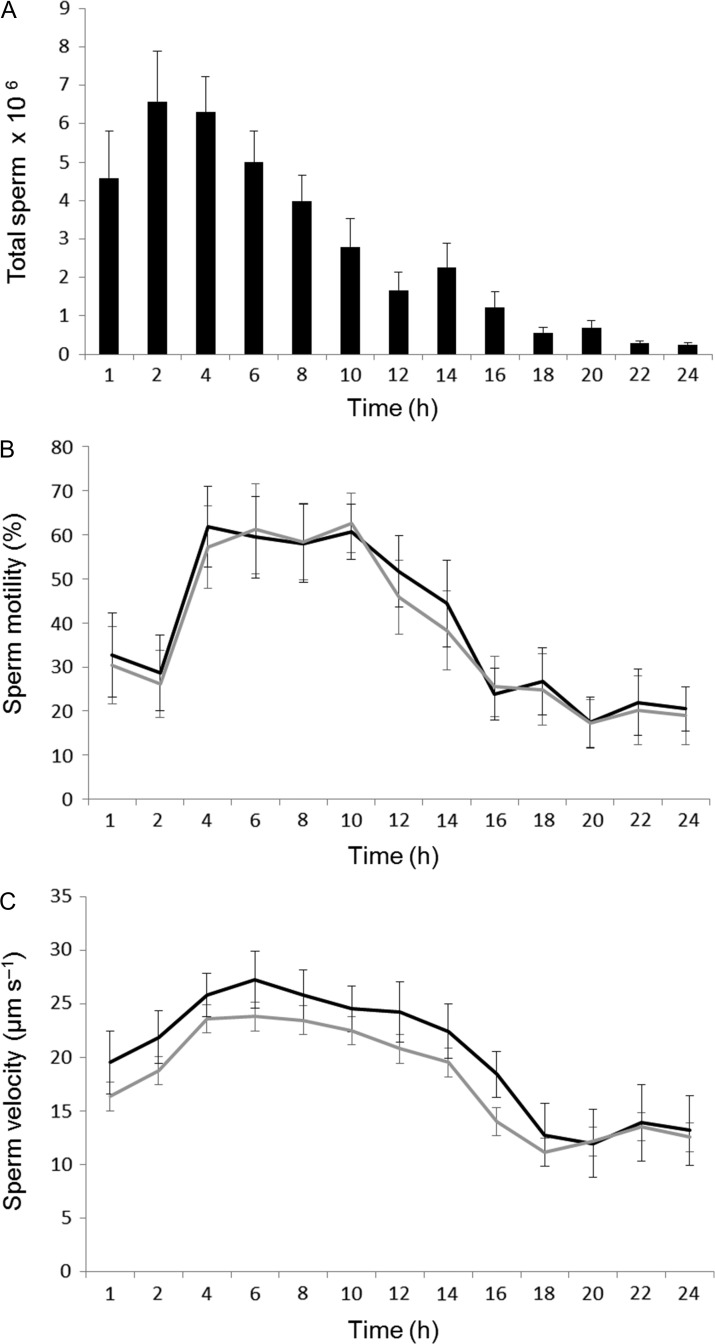
Effect of sampling time on (**A**) total number of sperm released, **(B**) percentage sperm motility and (**C**) sperm velocity (VCL) over a 24-h period post-administration of 40 IU g^−1^ hCG. Data are shown as the mean ± SEM (*n* = 10). Lines depict (**−**) undiluted spermic urine samples and (**−**) diluted spermic urine samples.

The total number of sperm released in response to the administration of 40 IU g^−1^ hCG differed significantly over time (LME; *F*_13_ = 12.131, *P* < 0.0001), with peak sperm release occurring between 1 and 6 h and steadily declining between 8 and 24 h post-hormone administration (Fig. 4a). Percent sperm motility differed significantly over time (LME; *F*_13_ = 31.360, *P* < 0.0001), but was not significantly affected by dilution treatment (LME; *F*_1_ = 0.705, *P* = 0.402). Percent sperm motility (58.1–62.7%) was highest between 4 and 10 h post-hormone administration decreasing to below 25 % by 20 h (Fig. 4b). Sperm velocity differed significantly over time (LME; *F*_13_ = 28.964, *P* < 0.0001), and was also significantly affected by dilution treatment (LME; *F*_1_ = 8.537, *P* = 0.004) . Sperm velocity was highest between 4 and 12 h post-hormone administration (24.3–27.2 μm s^−1^), decreasing to below 14 μm s^−1^ by 18 h (Fig. 4c). Diluting the sperm suspension consistently resulted in lower sperm velocity (Fig. 4c).

## Discussion

To date, few studies have quantified the efficacy of multiple doses of both GnRH-a and hCG at inducing amphibian sperm-release to ascertain optimal spermiation protocols. Instead, researchers have predominantly adopted a ‘trial-and-error’ approach, which has slowed the application of reproductive technologies for threatened species recovery. Results from the present study demonstrate that spermiation can be successfully induced during the natural breeding season of the critically endangered Booroolong frog following the administration of both GnRH-a and hCG, but that these hormones are not equally effective. The optimal sperm-release response following administration of GnRH-a was achieved at a dose of 0.5 μg g^−1^, with 82% of males releasing an average of 3.5 × 10^6^ sperm. The total number of sperm released peaked at the lowest dose and declined with increasing GnRH-a concentration. In contrast, the total number of sperm released in response to hCG increased with increasing dose, with sperm release peaking at 25.1 × 10^6^ in response to the administration of 40 IU g^−1^ hCG. Importantly, the response of male Booroolong frogs to the administration of hCG was consistent (100% response at all doses) and sperm output was greater in response to all hCG doses compared to the optimal concentration of GnRH-a.

Results from our study emphasise the importance of empirically testing the efficacy of GnRH-a and hCG at inducing spermiation in amphibians, adding to a growing literature indicating that there are species-specific differences in the sperm-release response triggered by exogenous hormones. Recent research suggests that species specificity in the optimal hormone type may be predicted by phylogeny, with species from the anuran families Myobatrachidae and Ranidae reportedly responding better to GnRH-a (Byrne and Silla, 2010; Calatayud *et al.*, 2016; Silla and Roberts, 2012; Uteshev *et al.*, 2012), while a number of species from the Limnodynastidae and Bufonidae families respond better to hCG (Kouba *et al.*, 2012; Silla and Roberts, 2012). The present study is the first to quantify spermiation in response to both GnRH-a and hCG in a species from the family Pelodryadidae. Studies with other species within this family (*L. aurea*, *L. caerulea* and *L. chloris*) have similarly shown that males release high numbers of sperm in response to hCG (Clulow *et al.*, 2018), however, experimental replication was low (*n* = 1–7) and the administration of GnRH-a was not tested. As a result optimal spermiation protocols have not been established for these species, and further research is required to ascertain whether species from the family Pelodryadidae consistently respond better to hCG. Confirming phylogenic differences in hCG and GnRH-a efficacy will allow protocol refinement for novel species to bypass the hormone testing stage. Protocol refinement can then focus on improving sperm quantity and quality through the establishment of hormone dose curves and identification of optimal sample collection times.

It is important to highlight our finding that spermiation improved with increasing hCG dose, but decreased with increasing GnRH-a dose. These results suggest that the highest hCG dose administered did not exceed optimum concentrations. In contrast, spermiation responses exhibited following GnRH-a administration peaked at the lowest dose, and when supraoptimal doses were administered spermiation declined. Exceeding optimal concentrations of GnRH-a is known to result in pituitary desensitization and down-regulation of GnRH-receptors, which leads to a reduction of LH synthesis and release and reduced gonadal stimulation (Silla and Byrne, 2019). Recently, there has been interest in trialling the use of GnRH-a in combination with dopamine antagonists (DA) in an attempt to enhance the efficacy of GnRH-a (Della Togna *et al.*, 2017; Nascimento *et al.*, 2015; Vu *et al.*, 2017). To date, however, there is no evidence that DA potentiate the effects of GnRH-a if optimal doses have been appropriately established (Silla and Byrne, 2019). For example, the spawning success of neither *Lithobates pipiens* nor *Lithobates catesbeianus* is enhanced by the combined administration of GnRH-a and DA, with the administration of GnRH-a alone at optimal doses shown to be equally effective (Nascimento *et al.*, 2015; Vu *et al.*, 2017). Similarly, the spermiation response of *L. booroolongensis* is not improved by the administration of GnRH-a in combination with the DA domperidone or metoclopramide (Silla & Byrne unpublished data). To date, only a single amphibian study reports a benefit of the use of a DA (Della Togna *et al.*, 2017). Importantly, while the study on *Atelopus zeteki* tested a range of GnRH-a doses (1, 2 and 4 μg/g GnRH-a), researchers did not test the dose used in combination with DA (0.4 μg/g GnRH-a) in isolation (Della Togna *et al.*, 2017). As a result, it is unclear whether GnRH-a at a dose of 0.4 μg/g would have induced a comparable spermiation response in this species. Results from these studies highlight the importance of comprehensively testing reproductive hormones at a range of doses, with the establishment of dose-response relationships for individual hormones critical prior to trialling hormone cocktails.

In addition to the importance of identifying the optimal hormone type and dose to induce sperm-release in a given species, it is imperative to quantify time-dependent responses and identify optimal collection times post-hormone administration (Kouba *et al.*, 2012). This information is necessary to ensure sperm samples of the highest quantity and quality in order to maximise fertilisation success during artificial fertilisation trials, and/or enhance post-storage viability where sperm samples are to be stored via refrigeration or cryopreservation (Shishova *et al.*, 2011; Silla *et al.*, 2015; Uteshev *et al.*, 2015). Despite the importance of this information, the majority of spermiation studies conducted to date have only employed three sampling points within a 12-h period (e.g. McDonough *et al.*, 2016; Silla, 2010; Silla and Roberts, 2012; Uteshev *et al.*, 2013), with few studies extending collection times beyond 12 h post-hormone administration, or collecting samples at more than six time periods (see Byrne and Silla, 2010; Della Togna *et al.*, 2017; Kouba *et al.*, 2012). The present study represents the most comprehensive sampling regime employed to date (13 collection times over a 24-h period), and clearly demonstrates time-dependent effects on total sperm number, sperm motility and velocity. Interspecific variation in the timing of responses to hormone treatment may reflect interspecific differences in mating system structure and associated differences in testes size, capacity for sperm production, and basal levels of circulating androgens (Silla and Byrne, 2019). Accordingly, optimal collection times are likely to vary significantly between species, and this is an area that requires research attention (Silla and Byrne, 2019).

In addition to species-specific differences in optimal hormone-induction protocols, it is important to note that amphibians display strong sex-specific variation. Male and female conspecifics differ considerably both in regard to optimal hormone types/doses, as well as peak collection times PA. While gamete-release in male amphibians is best achieved via the administration of a single hormone/dose, females can be more difficult to induce and typically require low-dose priming injections prior to the administration of a higher ovulatory dose and/or a combination of hormone types to achieve optimum results (Browne *et al.*, 2006; Byrne and Silla, 2010; Calatayud *et al.*, 2015; Silla, 2011). Such differences in hormone-induction protocols are likely to arise due to differences in gamete-maturation rates between the sexes, in addition to differences in the relative importance of FSH secretion in regulating gamete maturation and release. Specifically, gamete maturation is slower in females and the hypothalamic synthesis and release of high levels of both FSH and LH are required to promote vitellogenesis, oocyte maturation and ovulation (compared to males, which rely primarily on LH alone as the primary driver of spermatogenesis and spermiation; Vu and Trudeau, 2016). So, while the present study effectively identified the optimal hormone type/dose and collection period PA for male Booroolong frogs, these protocols are unlikely to result in the peak gamete-release response of female conspecifics. As such, an important next step will be to refine hormone-induction protocols for female Booroolong frogs. This knowledge will provide a platform to maximise artificial fertilisation outcomes.

## Conclusions

Reproductive technologies have enormous potential to contribute to CBPs by manipulating the neuroendocrine system of breeding stock in order to circumvent the behavioural and physical impediments to natural reproduction that captive amphibians often encounter. In recognition of this potential, reproductive technologies are being increasingly used to enhance the propagation and genetic management of threatened amphibians; however progress has been slow, which to a large extent, has been the result of trial-and-error approaches to protocol refinement. Herein, we report that both GnRH-a and hCG can be effectively used to induce spermiation in the critically endangered Booroolong frog, with a dose of 40IU hCG inducing 100% of males to release large quantities of viable sperm. We also identify the peak period of sperm collection post-hormone administration and highlight the importance of quantifying time-dependent responses on a species-specific basis. The present study represents the most comprehensive investigation of hormone-induced spermiation in an amphibian to date, both in regard to hormone treatments and sampling periods PA. Further research is required to extensively evaluate optimal hormone-induction protocols for a greater number of threatened amphibian species to expedite the incorporation of reproductive technologies into CBPs and boost global conservation efforts.
